# Drug Treatment of Cancer Cell Lines: A Way to Select for Cancer Stem Cells?

**DOI:** 10.3390/cancers3011111

**Published:** 2011-03-04

**Authors:** Ilaria Chiodi, Cristina Belgiovine, Francesca Donà, A. Ivana Scovassi, Chiara Mondello

**Affiliations:** Institute of Molecular Genetics, CNR, via Abbiategrasso 207, 27100 Pavia, Italy

**Keywords:** cancer, stem cells, drug resistance, chemotherapy

## Abstract

Tumors are generally composed of different cell types. In recent years, it has been shown that in many types of cancers a subset of cells show peculiar characteristics, such as the ability to induce tumors when engrafted into host animals, self-renew and being immortal, and give rise to a differentiated progeny. These cells have been defined as cancer stem cells (CSCs) or tumor initiating cells. CSCs can be isolated both from tumor specimens and established cancer cell lines on the basis of their ability to exclude fluorescent dyes, express specific cell surface markers or grow in particular culture conditions. A key feature of CSCs is their resistance to chemotherapeutic agents, which could contribute to the remaining of residual cancer cells after therapeutic treatments. It has been shown that CSC-like cells can be isolated after drug treatment of cancer cell lines; in this review, we will describe the strategies so far applied to identify and isolate CSCs. Furthermore, we will discuss the possible use of these selected populations to investigate CSC biology and develop new anticancer drugs.

## 1. The Cancer Stem Cells Concept

Cancers are composed of heterogeneous cell populations, among which putative cancer stem cells (CSCs), or tumor initiating/propagating cells, have received a great deal of attention in recent years. Cancer stem cells are defined on the basis of three main characteristics: (1) a selective tumorigenic capacity; (2) self-renewal and differentiation, that is the ability to sustain growth of heterogeneous cancer tissues, being able to recreate the different populations observed in the tumor; (3) expression of specific surface markers, allowing their reproducible selection [1].

The theory of CSCs implies that a large number of tumor cells have to be injected into immunocompromised mice to get a tumor, because only a limited number of them is endowed with tumorigenic potential; in contrast, injection of very few “selected” CSCs would be sufficient to generate a tumor. Evidence supporting this concept was first obtained in hematological tumors, then in breast and brain cancers, followed by many other solid tumors [2–5]. Recent investigations have shown that the fraction of propagating tumor cells is not necessarily small, it can be very high as in melanoma [6], and can vary with the stage of malignant progression [7–9].

CSCs can be selected on the basis of the expression of a repertoire of surface markers, including CD44, CD24, CD133 and EPCAM (epithelial cell adhesion molecule) [10]; aldeyde dehydrogenase I (ALDHI) is a marker typically expressed in breast CSCs [11]. Another characteristic distinguishing tumor-initiating cells is the ability to exclude Hoechst 33342 dye. Dye excluding cells, named side population (SP), can be separated from the bulk of the tumor population by cell sorting. SPs isolated from different tumors showed a higher tumorigenic potential in immunocompromised mice compared to non-SP cells, suggesting that they were enriched in CSCs [12–15]. The SP phenotype is related to the overexpression of ATP-binding cassette (ABC) transporters that can pump several types of endogenous and exogenous compounds out of the cell, including metabolites and drugs [16,17]. This phenotype can explain, at least in part, another important feature of CSCs: the high resistance to chemotherapeutic agents [18]. Chemoresistance and radioresistance have obvious implications for cancer treatment [19,20] and can be additional markers for CSC selection, as will be deeply discussed in subsequent paragraphs of this review.

CSCs can also be selected from tumors through their ability to grow in spheres, also known as tumorspheres. This culture technique was originally developed to expand neuronal stem cells, when it was found that, from a single cell suspension, a small proportion of cells, representing undifferentiated multipotent neuronal cells, could grow in spheres in the absence of attachment to an exogenous substrate. In the spheres, between 4% and 20% of the cells were stem cells, while the others represented progenitor cells in different phases of differentiation [21,22]. Similarly, stem/progenitor cells were enriched from mammary cell populations on the basis of their ability to grow in spheres (mammospheres) [23]. Subsequently, sphere culture techniques were applied to cell populations from a variety of cancers, comprising brain cancers, colon cancers, breast cancers and melanoma, and successfully allowed the enrichment of cells with CSC features [24–30]. Spheres grown from breast cancer samples or breast cancer cell lines, for example, were more tumorigenic than the bulk of the population, showed an enrichment in CD44^+^/CD24^−^ and SP cells, and a higher resistance to radiation [23,29,30].

## 2. CSC Origin

The observation that some of the CSC features described above are shared with stem cells raised the hypothesis that CSCs derive from stem cells of the tissue in which the tumor develops. In myeloid leukemia, cells with tumorigenic potential express CD34 and are negative for CD38 (CD34^+^/CD38^−^), as well as normal blood cells characterized by the ability to repopulate the hematopoietic system of SCID (severe combined immunodeficient) mice, suggesting that tumor cells could derive from these precursors [3]. However, CSCs could also originate from differentiated cells that undergo dedifferentiation after the acquisition of oncogenic lesions and the engagement in neoplastic transformation.

To this regard, it is worth mentioning that terminally differentiated cells can be experimentally induced to acquire the pluripotency of embryonic stem cells (induced Pluripotent Stem-iPS-cells), clearly demonstrating that differentiated cells can undergo dedifferentiation [31,32]. Interestingly, some of the defined genes able to induce iPS cells are well-known oncogenes, or can be amplified in tumors (e.g., c-*myc* and *SOX2*) [31,33], while functional tumor suppressor genes, such as for example *p16*^*INK4a*^, can be a barrier to dedifferentiation [34,35]. These observations indicate that tumor cells with stemness marks may indeed arise from differentiated cells after the acquisition of genetic lesions.

In support of this notion, it was shown that committed hematopoietic cells could undergo neoplastic transformation, becoming able to initiate and maintain leukemias, after exogenous expression of leukemogenic fusion proteins [36,37]. Moreover, cancer cells with stem-like properties were generated from pre-malignant mammary epithelial cells after transformation with activated oncogenes [38]. Cells with the ability to induce tumors in immunocompromised mice were also obtained from primary fibroblasts established in culture from skin biopsies, either after spontaneous neoplastic transformation [39], or after transformation with specific tumor genes [40]. Whether or not these transformed cell populations contained cells with a stem-like phenotype is not known; however, if they contained CS-like cells, it has to be argued that cells such as fibroblasts can acquire stemness markers during transformation, if they did not, it would mean that also cells devoid of stemness indicators can be endowed with tumorigenic potential.

In epithelial cancers, tumor progression and the acquisition of metastatic features are strongly connected with epithelial-mesenchymal transition (EMT) [41]. In the tumorigenic cell population obtained by Morel *et al.* [38] from pre-malignant breast cells, the stem cell-like phenotype was associated with features reminiscent of EMT, establishing a link between CSCs and EMT. In this line, evidence was reported that induction of EMT could generate cells with properties of stem cells [42,43].

Taken together, the data reported so far suggest that there is a high degree of plasticity between CSCs and non-CSCs, with the possibility that CSCs generate non-CSCs and *vice versa*.

## 3. CSCs, Drug Resistance and Cancer Cell Lines

One of the main critical points in cancer treatment is the emergence of tumor recurrences after therapy. The high resistance to chemotherapeutic drugs of CSCs can be an important element in cancer relapse. If cancer therapies mainly target highly proliferating cells, it is likely that they can kill the bulk of tumors, rather than the CSCs, which are relatively quiescent and resistant to chemotherapeutic agents. A large body of evidence indicates that chemoresistance is associated with selection of more resistant CSCs and that more aggressive and refractory cancers contain more tumor-initiating cells [25,44]. Thus, future cancer therapies should combine agents able to target both non-CSCs and CSCs. Nevertheless, despite improving the fight against cancer by following this direction, it cannot be forgotten that tumor cells are genetically heterogeneous because of the accumulation of mutations and can undergo divergent clonal evolution [45]. This genetic instability must exist also in CSCs, which could thus present the same phenomenon observed in bulk tumor cell populations; that is the development of drug resistance [46].

Isolation of CSCs can be an important step for the validation of drugs directed against them. As mentioned in the first paragraph, CSCs can be isolated on the basis of specific surface marker repertoires, the ability to exclude fluorescent dyes or particular culture conditions; chemoresistance can be an additional tool to select CSCs. Selection can be performed both from tumor specimens and cancer cell lines.

Cancer cell lines can be established *in vitro* from tumor samples. Although it could be argued that during cancer cell line establishment, only CSCs emerge and give rise to the cell line, given the observation that only a limited number of cancer cells from a biopsy develop foci and grow [47], several studies have reported that only a fraction of cells in tumor cell lines exhibits a CSC phenotype [48]. How different cell populations can be maintained during culture propagation is matter of debate, because cells proliferating faster should out-compete cells with a lower growth rate; however, it might be that CSCs and non-CSCs are in reversible equilibrium and/or the more differentiated cells have a limited proliferation potential.

The first evidence that cancer cell lines grown in culture for many years contain cells with a CSC phenotype was obtained by Kondo *et al.* [49], who found that cell lines as diverse as glioma (C6), neuroblastoma (B104) and breast cancer (MCF7) cell lines and HeLa cells, all contained a small SP. Studying the C6 SP cells in more details, the authors found that their growth in the absence of serum, but in the presence of platelet-derived growth factor (PDGF) and basic fibroblast growth factor (bFGF), greatly enhanced the proportion of CS-like cells in the cell culture, with the formation of floating neurosphere-like aggregates. Moreover, C6 SP cells, but not non-SP C6 cells, were able to generate both SP and non-SP cells and to form tumors after injection into immunocompromised mice. Finally, C6 SP cells could differentiate both in neurons and glia, indicating that they had the characteristics of multipotent cells.

The analysis of phenotypic and genotypic traits of CSCs derived from glioblastoma cell lines grown in the presence of bFGF and epidermal growth factor revealed that they were more similar to primary tumors than cells grown in the presence of serum, indicating that CSC enriched populations could be a more reliable tool to study the biology of primary tumors than cell lines *in toto* [50].

CS-like cells were also isolated on the basis of specific CSC features from cell lines established from different types of tumors [49-65] (a review of CSCs derived from cancer cell lines is presented in Table 1, together with the isolation methods and their main features). Sajithlal *et al.* [60] developed an original approach to isolate CSCs from human breast cancer cell lines. These authors tagged four breast cancer cell lines with the green fluorescent protein (GFP) under the control of the OCT3/4 stem cell-specific promoter; the GFP-positive populations were highly enriched in cells with CSC features and gave rise to tumors when injected into immunocompromised mice. Interestingly, almost all tumor derived cells displayed CSC characteristics, suggesting that the OCT3/4 promoter blocked CSC differentiation, with a mechanism unknown so far, consenting to obtain stable CSC lines, highly useful for further investigations. Long-term cultures of CS-like cells from breast cancer cell lines could be established, confirming the relevance of tumor cell lines to study cancer initiating cells [29].

## 4. CSC Selection from Cancer Cell Lines after Drug Treatment

Although CSCs were originally described in myeloid leukemia [3], their separation from leukemic cells isolated from patients still represents a problem, due to their scarcity and the possible contamination with normal hematopoietic stem cells (HSCs). It has been recently reported that leukemic CSCs (LSCs, leukemic stem cells) can be easily enriched from leukemic cell lines through drug selection. Given that some features of LSCs are similar to those of normal HSCs, an isolation strategy taking advantage of this peculiarity was envisaged. A protocol originally developed to isolate HSCs was applied to the human leukemic cell line KG1a; this method is based on the fact that HSCs and LCSs are quiescent and thus insensitive to drugs acting on proliferating cells, such as the pyrimidine antagonist 5-fluorouracil (5-FU). By incubating KG1a cells with 5-FU (50 μg/mL for 4 days), a KG1a CD34^+^/CD38^−^ cell subpopulation, corresponding to CSCs, was isolated and further characterized with respect to CSC markers. The population was enriched in Hoechst 33342-excluding cells, showed a proliferative potential *in vitro* after drug removal and a low level of RNA synthesis, consistent with a quiescent state. Moreover, these cells showed a higher expression of the transporter ABCG2 compared to the progenitor cells [66]. This latter feature, alteration in ABC transporters, is not exclusive to CSCs, being a hallmark of drug-resistance [67].

Isolation of CSCs through drug selection has also been applied to numerous solid cancer cell lines. Breast cancer still represents a major health issue worldwide. So far, different strategies based on single step or prolonged incubation of breast cancer cell lines with drugs have been developed for the isolation of breast CSCs. Calcagno *et al.* [68] demonstrated that long-term exposure of the MCF7 breast cancer cell line to increasing concentrations of doxorubicin not only selected for resistant cells (MCF-7/ADR), but also for cells with a stem-like phenotype, being the CD44^+^/CD24^−^ population highly enriched among resistant cells. Microarray analysis of global gene expression profiles of parental cells compared to the doxorubicin-resistant subline identified not only a panel of drug-resistance overexpressed genes, as expected, but also a stem-like gene expression profile in MCF-7/ADR cells. Furthermore, these cells formed mammospheres, were more invasive and more tumorigenic in mice than parental cells. Interestingly, drug resistant cells showed typical features of cells that have undergone an epithelial-mesenchymal transition, confirming the parallelism between CSC phenotype and EMT. Although these observations should be confirmed studying additional drug resistant tumor cell populations, they suggest that prolonged drug treatment of breast cancer patients may result in the selection of chemotherapy-resistant cells with a CSC phenotype, which might be true cancer stem cells.

An intriguing approach to isolate CSCs from the human breast cancer cell line MCF-7 was recently applied, consisting of the use of nicotine; this compound was tested on breast cancer cell lines with the aim of investigating whether smoking could increase breast cancer risk [69]. Using a concentration of nicotine closely related to the blood concentration in cigarette smokers (10 nM–10 μM), an increase in stem cell population, identified as ALDH^+^ cells, was observed in MCF7 cells. This population forming mammospheres was characterized with respect to Notch signaling, which is involved in anchorage-independent growth [70]. It was found that the Notch pathway was activated in ALDH^+^ cells through a kinase cascade. This evidence could discourage ladies from smoking!

About 20% of breast cancers are characterized by the *Her-2* oncogene amplification (and the consequent overexpression of the HER2 protein), which is associated with an aggressive disease and early development of metastasis [71,72]. HER2^+^ patients are usually treated with trastuzumab (Herceptin®; Genentech Inc., San Francisco, CA, USA), a human monoclonal antibody directed to the extracellular domain of HER2, which induces antibody-dependent cell-mediated cytotoxicity in tumor cells [73]. However, it is now clear that after an initial promising response, very often tumor cells acquire immunoresistance and cancer relapses [74]. Among the many reasons for this deleterious phenomenon [75-78], the possible selection of CSCs by trastuzumab was taken into account. To address this point, immunoresistant cells were isolated from human breast cancer cell lines treated with trastuzumab, and further characterized with respect to CSC markers [79]. Cells preferentially surviving the treatment showed the ability to grow in spheres, increased clonogenicity *in vitro* and tumorigenicity *in vivo*; moreover, they displayed a CD44^high^/CD24^low^ CSC-like phenotype. HER2 was still expressed in selected cells, although at a lower level compared to parental cells, indicating that immunotherapy resistance was not due to lack of HER2 expression and that HER2 has probably a role in the maintenance of breast CSCs [80]. Sorting and further culturing of CD44^high^/CD24^low^ cells revealed that they could revert to the original CD44^high^/CD24^high^ phenotype, confirming their CSC-like nature and indicating that the immunoselection process actually selected for CSCs. When immunoselected MCF7 cells were expanded in culture, they were still enriched in CD44^high^/CD24^low^ but showed a higher expression of HER2; this evidence suggests that treatment with anti-HER2 antibodies of tumors that initially showed a good response could still be beneficial after relapse [79].

To deeper characterize metastatic breast cancers, the relevance of hypoxia, which is a critical factor to promote invasive tumor growth, has been addressed by developing a protocol to expose human breast cancer metastatic cell lines to repetitive cycles of hypoxia-reoxygenation [57]. Under these conditions, which mimic the situation occurring *in vivo* during tumor development and metastatic property acquisition, the authors observed that a cycling cell subpopulation could be expanded, having a stem-like phenotype. Moreover, these microenvironment-selected cells were able to form colonies readily, to induce tumors in immunocompromised mice and exhibited an EMT phenotype. These observations suggest that a stem-like subpopulation in the tumor could expand selectively in response to changes in the microenvironment. The possibility to use niche factors in the tumor microenvironment to select CSCs from cancer cell lines opens up new chances to study and characterize these cells.

As for another hormone-dependent tumor, that is ovarian cancer, a strategy for isolating and propagating CSCs through drug selection has been recently described [81]. Employing cisplatin and paclitaxel, two widely used chemotherapeutic drugs, a subpopulation forming non adherent spheres was isolated from the human ovarian cancer cell line SKOV3 and characterized with respect to tumorigenic potential, expression of cell surface antigens and of a panel of stem cell genes by microarray analysis. The self-renewing isolated sphere cells were found to display stem cell properties, (expressing high levels of several stem cell genes, such as *Nanog, Oct4, sox2, nestin, ABCG2, CD133* and *CD117*), to be highly tumorigenic and, remarkably, resistant not only to cisplatin and paclitaxel but also to adriamycin and methotrexate. This latter feature renders them a suitable tool to test chemotherapeutic protocols in order to identify an efficient strategy to kill them. In fact, as recently reviewed [82], CSC isolation and characterization from ovarian cancer is extremely important for defining the therapy against this malignancy still representing the most fatal gynecological disease. In particular, the isolation of ovarian CSCs may facilitate the search for the mechanism(s) of intrinsic drug resistance, possibly due to many factors, including resistance to apoptosis, altered drug efflux, quiescence and proneness to tumor progression [82].

Prostate cancer, another hormone-mediated tumor, can be promoted by chronic exposure to arsenic, a carcinogen that attacks the urogenital system [83]. Chronic *in vitro* exposure of human prostate cells to inorganic arsenic can induce their malignant transformation [84]. Studying arsenite-induced transformation of the RWPE-1 normal prostate cell line, Tokar *et al.* [85] showed that arsenite resistant cells were enriched in cells with stem cell features, such as the ability to grow as spheres, hyperexpression of anti-apoptotic proteins, as Bcl2, MT1 and MT2, and low expression of pro-apoptotic Bax and apoptotic caspases. Moreover, CSC-like/arsenite-resistant cells were altered in drug efflux, having an intrinsic high expression of the *ABCC1* and *GSTP1* genes. Since normal prostate stem cells are intrinsically more resistant to arsenite than mature cells, it was hypothesized that drug exposure could select for stem cells present in the population, which could in turn be more keen to undergo malignant transformation.

Going deeper into drug selection of CSCs, Levina *et al.* [86] characterized drug surviving cells (DSCs) after exposure of the H460 human lung cancer cell line to etoposide, cisplatin or doxorubicin. DSCs were enriched in SP cells, expressed several stem cell markers (e.g., CD133, CD117, OCT4 and nuclear β-catenin, which is believed to be a key player in stem cell self-renewal capacity), and had lost the expression of differentiation markers, such as cytokeratins 8/18. Moreover, DSCs were able to grow as spheres, and maintained the capacity of self-renewal and to differentiate. However, upon differentiation, cells lost stem cell features and acquired drug sensitivity, while growth in the presence of drugs prevented differentiation, thus preserving the enrichment in CSCs of the population.

These observations suggest that chemotherapy increases the proportion of CSCs and eliminates the more differentiated tumor cells. In the classical chemotherapeutic plan, consisting of drug administration in several cycles separated by three-week intervals, differentiated cells could be possibly restored during the period in the absence of the drug. However, although differentiated tumor cells are drug sensitive, a population exposed to drugs contains a higher proportion of CSCs than the original population, therefore subsequent chemotherapy cycles could lead to tumors more and more enriched in resistant CSCs, rendering hard to eradicate cancer.

Another important result obtained by characterizing CSCs isolated after drug selection of H460 lung cancer cells was the understanding of some of the CSC properties that make these cells highly tumorigenic and metastatic [86]. By comparing sonicated lysates of parental and CSC tumors generated in mice, Levina *et al.* [86] showed that CSC-derived tumors produced higher levels of cytokines, chemokines, angiogenic and growth factors, which could mediate autocrine and paracrine signals giving rise to tumor cell proliferation and migration. In particular, CSCs expressed two-to-three fold higher levels of angiogenic and growth factors such as VEGF, PDGF-VV, bFGF, IGFBP-β and HGF, as well as higher levels of VEGFR1 and FGFR2 receptors. Moreover, CSC-derived tumors contained increased levels of cytokine IL-6 and chemokine IL-8, together with high levels of IL-8 receptors CXCR1 and CXCR2. Chemokines and growth factors produced by tumor cells, binding to their cognate receptors on tumor and stroma cells, could provide proliferative and anti-apoptotic signals helping tumor cells to resist to chemotherapeutic treatments.

In this respect, it has been recently reported [87] that a subpopulation of breast CSCs overexpressed the IL-8 receptor CXRC1 and selective CXRC1 blockade in two breast cancer cell lines, by means of specific antibodies or of the small molecule inhibitor repertaxin, affected the proliferation of CSCs *in vitro* and in nude mice, where tumor development and metastasis formation were delayed [88]. Of note, CSCs lacking CXRC1 expression were able to undergo apoptosis via the activation of FasL/Fas signaling.

The poor prognosis of patients with glioma prompted many groups to investigate the nature of chemotherapy-resistance of this aggressive tumor. Temozolomide, a DNA alkylating agent that blocks cell cycle at the G_2_/M phase, is widely used in clinics against brain aggressive tumors [89]. Exposure of U87MG and T98G glioma cells to DNA-damaging and clinically relevant doses of temozolomide led to the selection from each cell line of populations in which SP cells were increased 8- and 5-fold, respectively [90]. These SP cells showed tumor initiating potential and overexpression of ABCG2, as well as of other drug transporters. Moreover, in SP cells after temozolomide treatment, the percentage of cells co-staining for Msi-1, a marker for self-renewal [91], and ABCG2 was increased, suggesting that ABCG2 expression in CSCs with self-renewal capability could be important for drug efflux, and thus for drug resistance. Interestingly, ABCG2 knockdown did not abrogate the SP response to temozolomide, suggesting that other drug transporters could be activated leading to chemoresistance. On the basis of these results, it can be speculated that cancer recurrence could contain SP cells selected *in vivo* after temozolomide treatment for the expression of a drug-resistance gene. Preliminary data reported by Chua *et al.* [90] indicated that the percentage of SP cells was actually greater in a patient with recurrent glioblastoma multiforme than in a patient with no history of chemotherapy or radiotherapy.

The isolation of CSCs from the glioblastoma U251 cell line was obtained through the treatment with the chemotherapeutic drug etoposide. After etoposide exposure, U251 CS-like cells showed a greater resistance to apoptosis and death than the bulk of the tumor cell population [92].

Within the frame of a frantic active search for new and innovative compounds potentially acting as chemotherapeutic drugs, it has been recently reported that the use of pharmacological inhibitors of poly(ADP-ribosylation) could represent a novel therapeutic strategy to kill cancer cells as well as to attenuate the inflammatory processes that characterize many disorders [93–95]. Indeed, powerful protocols based on the pharmacological inhibition of PARP (poly(ADP-ribose) polymerase) enzymes are already adopted in clinical medicine especially to cure BRCA-deficient cancers [96]. However, the long-term effects of such drugs remain to be fully explored; a cautionary note originates from the fact that PARP enzymes are actively involved in DNA repair, thus the inhibition of their activity could impair the damage response [97,98]. In this respect, it has been recently shown that prolonged (100 days) incubation of human osteosarcoma MG-63 cells with 3-aminobenzamide (3-AB), a classic poly (ADP-ribosylation) inhibitor, resulted in the irreversible selection (even after 73 passages) of 3-AB-resistant cell population with stem cell features, such as ability to form sarcospheres, high drug efflux capacity, and stem cell surface marker expression. Of note, these cells acquired the ability to grow indefinitely, possibly because of telomerase activation, and expressed high levels of the Hif-1α protein, which is involved in managing cancer hypoxic microenvironment [99]. This approach, based on the use of 3-AB, could be applied to other cancer model systems, in order to explore the possible selection of CSCs through the inhibition of DNA repair cellular functions.

As for the treatment of recurrent cancers, there is a strong interest towards naturally occurring plant-based remedies and dietary factors, which could represent an alternative to conventional chemotherapy [100]. However, sometimes their beneficial effects could not be associated to a precise mechanism of action, thus making it difficult to manage their use. Natural compounds have been tested for their ability to target the CSC population isolated from cancer cell lines [101]. For example, curcumin, which has multiple beneficial effects on health [102], proved to reduce the SP fraction in rat C6 glioma cells, thus suggesting its potential use in brain tumor treatment [103]. These data are in line with previous reports on the effect of other plant compounds, such as parthenolide [104,105], berberine [106] and cyclopamine [107], found to be active on CSCs. Of course, further experiments are needed to confirm this evidence and to define the phenotypic parameters affected by phytochemicals. Among non-toxic agents possibly targeting cancer stem cells, it is worthwhile mentioning metformin, an anti-diabetic drug, which selectively targets breast cancer stem cells and, used in combination with chemotherapeutic agents, blocks tumor growth and prolongs remission [108,109].

## 5. Conclusions and Future Perspectives

Standard chemotherapy kills most of the cells in a tumor; however, some cells can survive and may cause tumor recurrence, even several years after treatment of the first tumor. A large body of evidence indicates that these cells could be the CS/tumor initiating cells, which are therefore a very important target for chemotherapy. Although CSC research is still in its infancy and many questions are still open, the isolation and further characterization of CSCs opened up new perspectives in clinical oncology. It is clear by now that cells with CSC features can be isolated from cancer cell lines; this is an important chance to study CSCs because of the high number of cells that can be obtained from cell lines, suitable for extensive molecular investigations and the analysis of cellular features, including growth parameters, tumorigenic potential, and drug sensitivity. Isolation of CSCs from cancer cell lines after treatment with drugs can mimic what happens *in vivo* during chemotherapy, thus making selected cells particularly appropriate to investigate drug resistance mechanisms and test new therapeutic agents. A future challenge for the use of *in vitro* cultured CSCs will be the development of culture conditions favoring the maintenance of their stem-like properties and, ideally, simulating the growth and differentiation inhibitory signaling provided by the microenvironment. Moreover, further investigations on CSC genome instability and evolution during cancer progression will have to be carried out, to design new therapeutic strategies that could be appropriate to counteract the development of drug resistance.

## Figures and Tables

**Table 1.** Cancer stem cell (CSC) selection from tumor cell lines.

**Cell line**	**Cancer**	**Selection method**	**Characteristics of isolated CSCs**	**Reference**
C6	rat glioma	SP * sorting	increased expression of bFGF and PDGF	[49]
			increased invasion ability	
MCF7, BT474, 734B	breast	sphere formation	increased expression of stemness markers§	[54]
			increased tumor-initiation ability	
			karyotype alterations	
A549	lung	sphere formation	increased expression of stemness markers	[54]
			increased tumor-initiation ability	
			karyotype alterations	
Cal-51	breast	SP sorting	increased expression of mammary	[52]
			epithelial markers	
SKBR3	breast	sphere formation	reduced let7 expression	[51]
SKOV-3	ovary	sphere formation	increased expression of stemness markers	[54]
			increased tumor-initiation ability	
			karyotype alterations	
3AO	ovary	sphere formation	increased expression of stemness markers	[61]
			increased tumor-initiation ability	
			drug resistance	
JR8	melanoma	sphere formation	increased expression of stemness markers	[54]
			increased tumor-initiation ability	
			karyotype alterations	
Me204ADH, JR8ADH, Me14346ADH	melanoma	sphere formation	increased expression of stemness markers	[59]
			increased tumor-initiation ability	
SW480	colorectal	stemness markers	increased expression of stemness markers	[56]
			increased expression of metastatic markers	
			high metastatic ability	
HCT116, HT29	colorectal	stemness markers	increased expression of stemness markers	[62]
			increased tumor-initiation ability	
			increased invasion ability	
			change in morphology	
SK-ES-1	Ewing sarcoma	SP sorting	increased expression of stemness markers	[65]
			increased invasion ability	
			increased proliferation rate	
			increased colony-forming ability	
			drug resistance	
Hu9, MG63 OS99-1, SaOS-3	osteosarcoma	sphere formation	increased expression of stemness markers	[64]
MG63	osteosarcoma	ALDH expression	drug resistance	[55]
			increased sphere formation	
HPET	prostate	stemness markers	increased expression of stemness markers	[53]
			drug resistance	
SK-RC-42	kidney	sphere formation	increased expression of stemness markers	[63]
			increased tumor-initiation ability	
			reduced proliferation rate	
			reduced immune-system stimulation	
			drug/radio resistance	
HT1080	fibrosarcoma	ALDH expression	increased sphere formation	[55]
SMS-KCN SMS-KAN CHLA-122	neuroblastoma	SP sorting	increased expression of stemness markers	[58]
			increased proliferation rate	
			increased colony-forming ability	

* SP: Side Population

§ Stemness markers include cell surface markers, such as CD44, CD24, CD133, CD105 (in particular for CSC selection), and markers of stem cells, such as Oct3/4, Nanog, Stat3, Sox2, BMI1.
